# Understanding the Pathogenesis of Spondyloarthritis

**DOI:** 10.3390/biom10101461

**Published:** 2020-10-20

**Authors:** Aigul Sharip, Jeannette Kunz

**Affiliations:** Department of Biology, School of Sciences and Humanities, Nazarbayev University, Nur-Sultan 010000, Kazakhstan; Aigul.Sharip@nu.edu.kz

**Keywords:** spondyloarthritis, HLA-B*27, pathogenesis, inflammation, arthritogenic peptides, unfolded protein response, ERAP1, gut dysbiosis

## Abstract

Spondyloarthritis comprises a group of inflammatory diseases of the joints and spine, with various clinical manifestations. The group includes ankylosing spondylitis, reactive arthritis, psoriatic arthritis, arthritis associated with inflammatory bowel disease, and undifferentiated spondyloarthritis. The exact etiology and pathogenesis of spondyloarthritis are still unknown, but five hypotheses explaining the pathogenesis exist. These hypotheses suggest that spondyloarthritis is caused by arthritogenic peptides, an unfolded protein response, HLA-B*27 homodimer formation, malfunctioning endoplasmic reticulum aminopeptidases, and, last but not least, gut inflammation and dysbiosis. Here we discuss the five hypotheses and the evidence supporting each. In all of these hypotheses, HLA-B*27 plays a central role. It is likely that a combination of these hypotheses, with HLA-B*27 taking center stage, will eventually explain the development of spondyloarthritis in predisposed individuals.

## 1. Introduction

Spondyloarthritis (SpA, also called spondyloarthropathy) is a group of inflammatory diseases of the joints and spine with various clinical manifestations. Five major subtypes of SpA are recognized on the basis of the classification criteria proposed by the European Spondyloarthropathy Study Group (ESSG) [1]. These subtypes include ankylosing spondylitis (AS), reactive arthritis (ReA), psoriatic arthritis (PsA), arthritis associated with inflammatory bowel disease (SpA-IBD), and undifferentiated spondyloarthritis (uSpA) [2,3]. More recently, a classification into axial and peripheral diseases has been proposed. The main clinical manifestations of SpA are inflammatory pain, and stiffness and swelling of the joints and spine. In addition, peripheral arthritis, enthesitis, dactylitis, sacroiliitis, and chronic inflammatory bowel disease (IBD) are often observed [3,4]. Less common symptoms are inflammation of the eye or GI tract, and hearing loss [5].

The exact etiology and pathogenesis of spondyloarthritis are still unknown. However, several lines of evidence indicate that genetics plays an important role in individuals’ susceptibility, while environmental factors, resulting in infections and gut dysbiosis, also contribute to SpA pathogenesis. Various studies demonstrated that microorganisms play a role in triggering the disease. 

**Figure 1:**
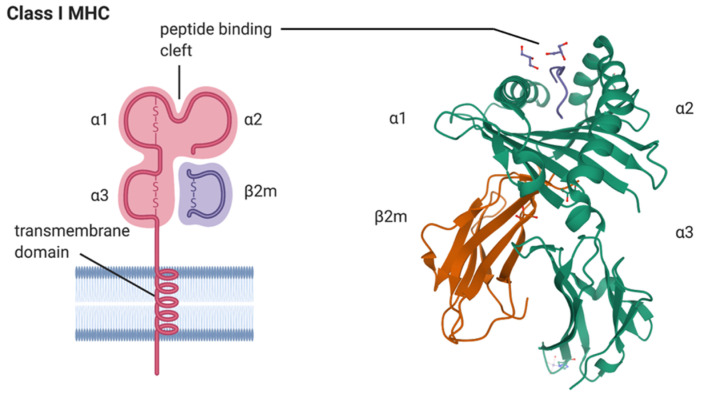
A model depicting the major histocompatibility complex (MHC) class I HLA-B*27 heavy chain in complex with beta 2-macroglobulin (β2m) is shown on the left (created with BioRender.com). The alpha 1 (α1), alpha 2 (α2), and alpha 3 (α3) domains of the HLA-B*27 molecule and the peptide binding cleft composed of α1 and α2 domains of HLA-B*27 are indicated, as is the transmembrane domain inserted into the lipid bilayer of the plasma membrane. A ribbon model of the crystal structure of the human class I MHC molecule HLA-B*2705 heavy chain (green) bound to nona-peptide m9 (blue), and in complex with β2m (orange), is shown on the right. Orientation: cell surface at top of picture. The image was sourced from the Research Collaboratory for Structural Bioinformatics (RCSB; rcsb.org) Protein Data Bank (PDB) entry ID 1JGE (DOI:10.2210/pdb1JGE/pdb; [9]) generated by PISA [10].

Genetically, SpA is strongly associated with the major histocompatibility complex (HMC) class I antigen, HLA-B*27. For example, more than 95% of patients with AS possess HLA-B*27, whereas its frequency among the general population is less than 10%. The risk of developing AS is as high as 5–7% in HLA-B*27-positive individuals [5]. HLA-B*27 consists of an alpha chain encoded in the B locus of the MHC region on chromosome 6, and a non-MHC encoded beta chain, β_2_ microglobulin (Figure 1). The primary function of HLA-B*27 in complex with β2-microglobulin is to present short antigenic peptides for recognition to cytotoxic T lymphocytes (CTLs; Figure 1). The ability of HLA-B*27 to bind a unique set of peptides may be the underlying reason for its disease association with SpA [6,7]. Three unique features of HLA-B*27, including peptide binding specificity, a tendency to misfold, and the predisposition for forming heavy chain homodimers may contribute to disease pathogenesis [8]. Each of these features have been suggested independently to play a role in the pathogenesis of SpA.

HLA B-27 is a highly polymorphic molecule of which, currently (August 2020), more than 223 subtypes are known (https://www.ebi.ac.uk/ipd/imgt/hla/allele.html). The high number of polymorphisms, in combination with heterozygosity, give a selective advantage to the immune system against the diversity of microorganisms and their antigens. However, extreme polymorphism and potential mutations in the major histocompatibility complex (MHC) increase the chance of developing autoimmune diseases [11]. Thus, these molecules play an essential role in the regulation of the host’s immune response, by initiating tolerance, and CTL or helper T cell responses, by presenting peptides to T cell receptors [12,13]. Among the HLA-B*27 alleles, HLA-B*2705 and HLA-B*2704 are the most strongly associated with SpA, whereas, HLA-B*2706 and B*2709 are weakly associated [14]. It is well known that the prevalence of SpA varies strongly between ethnic groups and populations, this can partly be explained by differences in the prevalence of HLA-B*27 [3,15]. The prevalence of AS varies from 0.2% to 0.7% among European populations, while higher frequencies of AS have been reported in populations from Siberia, Alaska, and Scandinavia [15]. 

The interaction between bacteria and HLA-B*27 alleles plays an important role in the hypotheses of SpA pathogenesis [16]. The evidence that ReA is triggered by *Chlamydia trachomatis* (genitourinary infections) or by other gram-negative enterobacteria, such as *Shigella, Salmonella, Yersinia,* and *Campylobacter* species, provides a solid background for these hypotheses [17]. In addition, inflammatory arthritis frequently develops following infection with enteric organisms, such as *Clostridium difficile, Brucella,* and *Giardia*. The identification of microbial antigens in the synovium of ReA indicates that those antigens can be crucial for ongoing joint inflammation [18]. The fact that some SpA types can be triggered by bacterial infections proves the importance of these organisms in SpA pathogenesis. Therefore, inappropriate immune responses to commensal bacteria or alterations in gut microbiota were also proposed to be factors in SpA development.

## 2. Models of SpA Pathogenesis: Hypotheses

The exact pathogenetic role of HLA-B*27 is unknown [19]. There are, however, some predominant theories explaining the role of the HLA-B*27 allele in SpA and disease pathogenesis, including the arthritogenic peptide hypothesis, misfolding protein hypothesis, as well as the hypothesis of the cell-surface HLA-B27 homodimer [20]. 

Numerous hypotheses have been suggested regarding the role of HLA-B*27 in the pathogenesis of AS. These are mainly based on rodent animal models. One of the oldest, and major, theories for explaining HLA-B*27 pathogenicity, is the so-called “arthritogenic peptide” hypothesis. This hypothesis proposes that the presentation of unique sets of antigenic peptides, either self- or bacteria-derived, by HLA-B*27 alleles to CD8^+^ T cells, results in cross-reactivity and an HLA-B*27–restricted cytotoxic T cell response. This response subsequently triggers a harmful CD8^+^ T cell-mediated response in joints and other affected tissues [15,21,22,23,24]. The second hypothesis, the “unfolded protein response”, suggests that HLA-B*27 tends to fold slowly and often misfolds. The misfolded HLA-B*27 molecules accumulate in the endoplasmic reticulum (ER), where they induce a stress response that leads to an unfolded protein response (UPR) and autophagy [22,23,24]. The upregulation of UPR genes subsequently activates the production of pro-inflammatory cytokines such as IL-17, IL-23, and IFN-γ [23]. Another hypothesis that explains the role of HLA-B*27 in SpA pathogenesis is the “formation of homodimers” hypothesis. According to this hypothesis, HLA-B*27 heavy chains homodimerize at the cell surface, where they act as pro-inflammatory ligands for humoral or cell-mediated autoimmune responses [14,22]. These homodimers are capable of binding to certain killer cell immunoglobulin-like receptors (KIRs), which are expressed on natural killer cells and T cells, and this leads to the upregulation of the pro-inflammatory cytokine IL-17 [24].

In addition, a hypothesis that has gained much attention focuses on the enzymes that trim peptides for loading onto MHC class I molecules, and presentation to T and NK cells. The gene with the second strongest SpA association, endoplasmic reticulum aminopeptidase 1 (*ERAP1*), trims peptides to optimal sizes for antigen presentation. Loss-of-function alleles of *ERAP1* lead to abnormal HLA-B*27 presentation and accumulation of intracellular free heavy chains, which can result in changes in peptide processing with pathological consequences [25,26]. Finally, there is mounting evidence pointing to the roles of non-genetic contributors to SpA and joint inflammation, specifically, the intimate relationship between infections, HLA-B*27, and gut dysbiosis in SpA. Many studies indicate a link between intestinal bacterial composition, in particular gut dysbiosis, and SpA. In this review, we will discuss the hypotheses to explain the disease mechanisms of SpA pathogenesis. We will also propose a hypothesis that combines the previously proposed hypotheses.

### 2.1. Arthritogenic Peptide Hypothesis

The arthritogenic peptide hypothesis is one of the oldest hypotheses explaining the association between HLA-B*27 and SpA [27]. It proposes that some HLA-B*27 subtype alleles, due to their characteristic amino acid residues, bind a specific arthritogenic peptide that is recognized by CD8^+^ T cells [28]. This hypothesis assumes that the natural ligands of HLA-B*27 are target antigens of autoimmune T cells, induced by cross-reactive bacterial peptides. Peptide presentation by HLA-B*27 is affected by subtype polymorphisms that affect the peptide-binding groove [29]. Consequently, a set of unique antigenic peptide epitopes, with key conserved anchor residues, are recognized by specific HLA-B*27 alleles. The arthritogenic peptide hypothesis is based on the peptide binding specificity of HLA-B*27, the concept of molecular mimicry, and the idea that activation of T cell responses by an external antigen may result in autoimmunity. The initial pathogenic event in SpA could be the activation of HLA-B*27 by CTL responses against bacterial peptides. Cross-reaction of some activated CTL with a self-peptide would subsequently lead to autoimmune tissue damage and inflammation [30].

Various studies support the arthritogenic peptide hypothesis. For instance, HLA-B*27-restricted CD8^+^ T cell responses specific for *Salmonella* or *Chlamydia* have been identified in patients who developed reactive arthritis, following infections with these pathogens [31,32]. Furthermore, B27-restricted CD8^+^ CTL were found in the synovial fluid of ReA patients [33,34]. A set of peptides derived from enteric organisms were identified that bound to, and have sequence homology with, HLA-B*27 [35]. AS patients also have a higher probability of having autoantigenic antibodies directed against *Klebsiella* that are cross-reactive against B27, in comparison to HLA-B*27 positive but healthy individuals [36]. Moreover, investigation of the HLA-B*27 peptidome identified more than a thousand peptides from HLA-B*27^+^ cell lines, half of which were found to tightly bind to HLA-B*27 alleles. Twenty-eight of these peptides were considered to be arthritogenic peptides as they were derived from cartilage or bone proteins that had high homology with proteins from enteric bacteria. These findings suggest that these antigenic peptides can induce a T cell mediated immune response that results in SpA pathogenesis [27,37]. Along with bacterial peptides, viral peptides could also contribute to SpA pathogenesis. In some SpA patients, cross-reactive CD8^+^ T cell responses, which recognized both an epitope from Epstein-Barr virus (EBV) and a self-peptide derived from the vasoactive intestinal protein receptor (VIPR), were identified [38]. However, EBV infection is not recognized as a SpA trigger and these findings warrant further investigation. Major support for the arthritogenic peptide hypothesis comes from studies of HLA-B*27 alleles and their differential association with SpA pathogenesis. For example, some alleles such as HLA-B*2705, HLA-B*2702, and HLA-B*2704 are strongly associated with the disease, whereas some other alleles (HLA-B*2706 and HLA-B*2709) were not, or only weakly, associated with disease pathogenesis [39].

Nevertheless, conclusive evidence that directly implicates molecular mimicry as a mechanism underlying the pathogenesis of SpA is still lacking [22]. Indeed, analysis of the HLA-B*27 peptidomes from SpA-related and unrelated alleles revealed that no clear distinction in qualitative differences between these groups was found, indicating that binding preferences of HLA-B*27 alleles do not solely explain disease association [40,41].

### 2.2. The Unfolded Protein Response Hypothesis

The unfolded protein response (UPR) hypothesis is another theory explaining the role of HLA-B*27 in SpA pathogenesis. Compared with other MHC molecules, HLA-B*27 tends to slowly fold or even misfold in the endoplasmic reticulum (ER), and to accumulate in the ER for extended periods of time, which may have implications for pathogenesis [42]. The accumulation of misfolded forms of HLA-B*27 in the ER leads to ER stress that induces the UPR, which is a homeostatic mechanism that aims to return the cell to its normal state [43]. 

UPR suppresses protein translation and upregulates ER chaperone molecules such as immunoglobulin heavy-chain-binding protein (BiP) and endoplasmic reticulum-localized DnaJ 4 (ERdj4). It also activates transcription factors such as CCAAT-enhancer-binding protein homologous protein (CHOP) and increases the production of pro-inflammatory cytokines (IL-23, INF-β, and IL-1) [44]. Indeed, UPR affects cytokine production on multiple levels, from stimulation of surface receptors to activation of inflammatory signaling pathways, in particular the IL-23/IL-17 signaling pathway [45]. Autophagy, induced by ER stress and UPR, also increases the production of IL-23 [46]. 

Misfolded HLA-B*27 molecules in the ER are disposed of by ER-associated degradation (ERAD), therefore, slow folding or accumulation of misfolded HLA-B*27 increases levels of ERAD, and stimulates UPR and autophagy, in particular during inflammation, when HLA-B*27 production is high [46,47]. The slow-folding kinetics and partial misfolding of HLA-B*2705 further lead to the activation of NF-kB, which leads to increased production of pro-inflammatory cytokines, such as TNF, IL-1, and IL-6. 

In support of the UPR hypothesis, transgenic rat models of AS, and characterization of synovial tissues of SpA patients, showed that HLA-B*27 misfolding and UPR occur in the gut and synovial tissues [48]. Bone marrow-derived macrophages from HLA-B*27 transgenic rats showed evidence of HLA-B*27 misfolding after cytokine stimulation, and this was associated with stimulation of IL-23 production [49,50]. These HLA-B*27 transgenic rats had functional alterations in the number of cell populations, which in turn can be correlated with HLA-B*27 misfolding. 

In particular the heavy chain β2 microglobulin is prone to misfolding [49,51,52,53] leading to the activation of the UPR [54]. Interestingly, studies that aimed to link the extent of β2 microglobulin misfolding to UPR activation, inflammation, and SpA development in HLA B27-transgenic rats demonstrated that the introduction of additional copies of the human β2-microglobulin gene, which reduced HLA-B*27 misfolding and UPR activation, led to an increase in the incidence and severity of arthritis [55], rather than the expected decrease. Interestingly, these studies led to the creation of rat strains with arthritic disease that closely resembled human spondylarthritis but showed no evidence of gut inflammation, indicating that spondylarthritis and IBD, particularly Crohn’s disease, may involve separate disease mechanisms [55]. These studies challenged the view that HLA-B*27 contributes to spondylarthritis through misfolding, formation of heavy chain β2 microglobulin dimers, and activation of the UPR, but supported the idea that UPR triggering is associated with inflammatory disease mechanisms that can lead to gut inflammation in SpA patients [55]. Consistent with this view, studies of tissues of AS patients did not suggest that UPR and ER stress was directly associated with inflammation and HLA-B*27 misfolding [56,57]. Thus, HLA-B*27 was found to lead to misfolding and ER stress in both in vitro and animal studies; however, currently, there is no evidence of the involvement of this process in SpA pathogenesis in humans.

The development of reactive arthritis (ReA) is associated with prior *Salmonella enterica* infection in the presence of HLA-B*2705. A recent study showed that *Salmonella* activates the UPR, leading to increased *de novo* lipid synthesis for ER biogenesis and membrane expansion under ER stress conditions [58]. While *Salmonella* can activate the UPR by itself, HLA-B*27 misfolding and pre-existing UPR activation are associated with enhanced Salmonella replication [58]. These data provide a potential connection between pathogenic properties associated with HLA-B*27 misfolding and cellular processes that may contribute to ReA pathogenesis. These studies further raise the question of whether HLA-B*27 misfolding and triggering of the UPR may allow for conditions that are favorable for *Salmonella* persistence in the gut and thereby play a role in HLA-B*27-associated gut inflammation.

Although several studies support the role of HLA-B*27 misfolding and assembly in the activation of the UPR and inflammation, the actual underlaying pathogenic mechanisms, and effect on SpA pathogenesis appear to be quite complex. It was demonstrated that, in M-CSF-derived macrophages from AS patients, UPR pathway genes (CHOP and BiP) and IL-23 are upregulated in comparison to healthy controls [59]. Recently, it was suggested that autophagy, but not UPR, is responsible for the regulation of IL-23 expression in the gut of AS patients [57]. This hypothesis is further supported by the observation that disease severity is unaffected by the lack of functional CD8^+^ T cells, but requires CD4^+^ T cells in HLA-B*27 transgenic rats [39,60,61]. 

Overall, the role of the ER stress response and the UPR in SpA pathogenesis remains incompletely defined, and a clear link between accumulation of misfolded HLA-B*27 and the production of proinflammatory cytokines remains to be demonstrated, as does the association between the pathogenic role of HLA-B*27 and the inflammatory IL-23/IL-17 axis. 

### 2.3. HLA-B*27 Homodimer Formation Hypothesis

The ability of HLA-B*27 to form disulfide-bonded homodimers is one of the unique characteristics of this molecule. These homodimers are expressed on the cell surface of a variety of cell types, including natural killer cells (NKs), CTLs, and B lymphocytes [14]. It has been suggested that homodimer formation is a sign of HLA-B*27 misfolding within the ER, and that the accumulation of misfolded protein potentially results in a pro-inflammatory stress response [42]. 

ER protein misfolding has a variety of biological effects, depending on the nature and quantity of the misfolded proteins, as well as on the severity of the defect. UPR activation can alter cytokine production, including increased production of pro-inflammatory cytokines IL-27, which is essential for SpA pathogenesis. Altered production of TNF and IFN-γ may play a crucial downstream role that contributes to the unique spondyloarthritis phenotype [8]. HLA-B*27 homodimers can facilitate the upregulation of the IL-23/IL-17 signaling pathway by two mechanisms: first, by increasing the production of Th17 cells, which in turn leads to production of IL-17/IL-23 [43], second, the binding of HLA-B*27 homodimers to KIRs and leukocyte immunoglobulin-like receptors (LILRs), which are expressed on NKs and T cells, also promotes the IL-23/IL-17 signaling pathway and inflammation [24,61].

There is much evidence supporting the HLA-B*27 homodimer hypothesis. Both KIRs and LILRs play crucial roles in immune responses, including the differentiation of macrophages and dendritic cells, T cell survival, and activation of regulatory T cells (Tregs) [62]. The KIR3DL2 receptor was shown to recognize HLA-B*27 homodimers, with higher affinity than normal HLA-B*27 heterodimers [20,63]. So far, seventeen KIR genes have been identified, and the interaction of KIR3DL2 with HLA-B*27 free heavy chains was shown to have pro-inflammatory effects in NK and T cells, and was linked to elevated Th17 expression in AS patients [64,65,66]. The binding of KIR3DL2 with HLA-B*27 homodimers was also observed to facilitate the survival and differentiation of CD4+ T cells in SpA patients. In addition, it increased production of pro-inflammatory cytokines, such as IL-17, TNF, and IFN-γ [20,62,67]. Moreover, in HLA-B*27 positive SpA patients, KIR3DL2^+^ NK cells and CD4^+^ T cells were increased in peripheral blood, and showed a higher level of cytotoxicity and IL-17 production than in HLA-B*27 negative SpA patients [63,68]. Thus, the binding of HLA-B*27 homodimers to KIR and LILR facilitates inflammation by enhancing the survival of NK and T cells, and affecting the differentiation of LILR-expressing antigen-presenting cells [15,39,62]. In addition, the arthritis-associated HLA-B*2705 allele was shown to form more cell-surface homodimers than the non-associated HLA-B*2709 allele [69].

Nevertheless, some research suggests that the formation of HLA-B*27 homodimers is not a major driver of SpA pathogenesis. The ability to form homodimers of eight common HLA-B*27 alleles (HLA-B*2702 to HLA-B*2709) was analyzed and no major differences were found between disease-associated and non-associated HLA-B*27 allotypes. In particular, HLA-B*27:03, an arthritis-associated HLA-B*27 subtype, had a reduced ability to form cell-surface homodimers [70]. These findings challenge the role of HLA-B*27 homodimer formation in SpA pathogenesis and suggest that other features of HLA-B*27 play a more central role in the disease mechanism.

### 2.4. The ERAP Polymorphism Hypothesis 

The genetic association of polymorphisms in the aminopeptidases, ERAP1 and ERAP2, with AS is the second strongest after HLA-B*27, and contributes approximately 15–25% of the population risk [25,71]. This association was first reported in the UK and the USA, and was validated in independent studies with other populations [23,72,73,74]. Together, HLA-B*27 and ERAP explain 70% of the genetic risk of developing SpA [75,76]. ERAP1 and ERAP2 are members of the zinc metallopeptidases that localize to the ER, and serve to trim peptides for loading and presentation by MHC I class molecules. Peptides displayed by MHC class I molecules are products of cellular protein degradation, either by the action of the proteasome [77,78,79] or, alternatively, by lysosomal degradation of proteins that were internalized through autophagy or phagocytosis [79,80,81]. Many of these peptides exceed the optimal length of 8–9 amino acids for presentation by MHC class I molecules and are transported into the ER via peptide transporters, called transporter associated with antigen processing (TAP1/TAP2) [82,83], and trimmed by ERAP1 and ERAP2 to a suitable length of 8–10-mer peptides, with appropriate anchor residues for MHC class I binding [84]. While ERAP1 and ERAP2 are related aminopeptidases, they differ in their peptide preference, trimming properties, and mode of association with SpA. 

ERAP1 is thought to be the main enzyme in the ER involved in peptide trimming, while ERAP2 appears to play a more minor role [85]. ERAP1 has two major functions. The first is its role in antigen cross-presentation, by trimming the N-termini of peptides in the ER to a suitable length to allow binding to MHC class I molecules, and presentation on the cell surface to CD8^+^ T cells or NK cells [85]. The second function of ERAP1 is the proteolytic cleavage of cytokine receptors, such as TNFR1, IL6R2, and IL1R2, that are expressed on the cell surface through receptor cleavage [72,86]. The shedding of cell surface receptors by ERAP1 can regulate the cellular immune response by modulating the receptor availability on the cell surface, which leads to a reduction of pro-inflammatory signaling [72,86]. Cleavage of the TNF receptor that was associated with an altered cellular cytokine response, and significant differences in mRNA expression of certain cytokines were observed in studies of AS patients with different *ERAP1* haplotypes [26,87]. However, while some *ERAP1* polymorphisms might be associated with SpA by increasing TNF receptor shedding [86], prevailing evidence suggests that shedding of cytokine receptors does not play a major role in SpA development [73,88]. 

ERAP1 preferentially generates peptides of 8–10 amino acid length from longer (more than 10 amino acids) peptides. ERAP1 will also act on 9-mer peptides and trim these to an even shorter length [89]. In this regard ERAP1 shapes the peptidome available for presentation, both by generating peptides of optimal length (8–10-mers) for acting as MHC class I ligands, and by the over-trimming of peptides, resulting in their elimination as potential MHC class I ligands. ERAP2 differs from ERAP1 through its preference for shorter substrates (7–8-mers) and its ability to trim these to even shorter peptides not suitable for MHC class I presentation. ERAP2 is produced at much lower levels when compared to ERAP1, and is even absent in 25% of humans that carry a specific *ERAP2* polymorphism [90]. In addition, rodents only express *ERAP1*, which limits the functional analysis of human *ERAP2* [90,91]. To date, ERAP2 has not been implicated in receptor shedding.

ERAP1 and ERAP2 also differ in their peptide specificities: ERAP1 prefers substrates containing hydrophobic residues (e.g., Leu) at the N-terminus and large hydrophobic residues at the C-terminus [92], whereas ERAP2′s aminopeptidase activity favors peptides with basic residues (Arg, Lys) in the N-terminal region, but has no preference for specific C-terminal residues [92]. Both aminopeptidases fail to cleave peptide bonds involving a Pro residue [92]. Therefore, ERAP1 and ERAP2 can have complimentary effects on the shaping of the antigenic repertoire.

The fact that the strong genetic association of AS with *ERAP1* was only found in HLA-B*27 positive individuals indicates that ERAP1 most likely interacts with HLA-B*27 within the antigen-processing and presentation pathway [38,71]. This suggests that abnormal peptide processing and antigen presentation is a crucial contributor to SpA pathogenesis [73,93]. Epistatic interactions with ERAP1 were also identified for Behçet`s disease, with HLA-B51 [94], and for psoriasis, with HLA-Cw6 [95]. These interactions suggest the possibility that similar HLA-driven pathogenic mechanisms underlay these diseases. ERAP2 was also genetically associated with psoriasis and AS development; however, there was no epistasis with HLA-B*27 [96,97,98,99].

While the exact mechanism behind the genetic association of AS with *ERAP1* is unclear, the systematic study of *ERAP1* loss-of-function mutations and polymorphisms suggests that *ERAP1* plays a key role in SpA pathogenesis, either through variation in the peptide repertoire bound to HLA-B*27 or through the generation of abnormal intracellular and extracellular forms of HLA-B*27, by affecting either ER misfolding and/or the export of pro-inflammatory B27 forms [100]. Indeed, disease-associated *ERAP1* alleles are loss-of-function variants, indicating that altered peptide processing and presentation are involved in disease development. Loss-of-function *ERAP1* polymorphisms affect HLA-B*27 heavy chain expression, dimerization, and folding [73,101], and reduce cell surface levels of HLA-B*27 homodimers [39,64]. These effects appear to be at least in part dependent on ERAP1′s enzymatic activity, because the loss of its trimming function similarly results in changes in the normal expression of HLA-B*27 and increases intracellular free heavy chain forms of HLA-B*27 in antigen-presenting cells [41,102,103]. More importantly, loss of ERAP1 function in humans and mouse models also leads to substantial changes in the MHC class I peptidome, indicating that it is important for shaping the MHC class I peptide repertoire [104].

Various *ERAP1* polymorphisms have been identified that are associated with SpA development [105]. For example, rs30187, a polymorphism that changes the amino arginine to the more enzymatically active lysine at position 528 (R528K), significantly increases disease risk [101]. This mutant variant alters peptide processing, thus supporting the notion that altered peptide characteristics contribute to disease development [101]. The effect of *ERAP1* polymorphisms on the susceptibility to SpA may vary among ethnic groups; therefore, identification of genetic variations in different populations could be important for understanding SpA pathogenesis [26,105]. For example, the *ERAP1* polymorphisms rs30187, rs27044, and rs2287987 were associated with AS susceptibility in a Polish population [106]. While, rs30187 and rs2287987 were also predominantly associated with the risk of developing AS in an Iranian cohort [26]. Interestingly, the polymorphism rs75862629 provided protection from AS in HLA-B*27 positive individuals in Sardinia, by regulating the expression of both ERAP1 and ERAP2 [107]. ERAP2 protein levels are generally not affected to the same extent by polymorphism as ERAP1. Nevertheless, *ERAP2* polymorphisms may also be associated with AS [27].

The relationship between ERAP1 and HLA-B*27 might be crucial for SpA development, as it was shown that the inhibition of ERAP1 leads to changes in cells with disease-associated HLA-B*2704 or HLA-B*2705, but not in cells with the non-associated HLA-B*2706 or HLA-B*2709 [23]. In particular, the association between *ERAP1* polymorphisms and the development of AS supports the arthritogenic peptide hypothesis, although additional effects, such as those on HLA-B*27 dimerization and surface expression, likely contribute.

The effects of ERAP1 or ERAP2 loss-of-function mutants or polymorphisms on the presentation of peptides by MHC class I molecules have been comprehensively analyzed in a number of studies that were covered in recent reviews [85,108,109]. As only MHC class I molecules loaded with peptide are able to exit the ER, and are transported to the plasma membrane, the complete absence of ERAP1 results in the reduction of MHC class I cell surface levels [110]. Most studies have revealed reduced MHC class I surface expression in situations of reduced ERAP1 function. This has been attributed to the instability of the cell surface MHC complexes bound to peptides, which are non-optimal substrates. Such peptides may accumulate, because the absence or downregulation of ERAP1 function alters the peptide reservoir available for display by MHC class I molecules, leading to a shift to longer peptides and the generation of “novel” peptides that would otherwise have been lost due to over-trimming [111]. Moreover, as HLA class I molecules can accommodate longer peptides in their peptide binding cleft [112,113,114], *ERAP1* polymorphisms that alter protein levels and activity result in the presentation of a different set of ligands in an HLA-B*27 background and, consequently, altered antigenicity. Consistent with the notion that altered peptide characteristics underlie disease pathogenesis, a loss-of-function ERAP2 variant that is associated with AS does not affect MHC class I surface levels, endoplasmic reticulum stress, or inflammatory cytokines production [98,115], but alters the peptidome available for MHC class I presentation [116,117].

Given that there is a shift to longer peptides due to abnormal peptide processing caused by altered ERAP1 expression or activity, some of these peptides may play a crucial role in the pathogenesis of SpA, by changing the antigen presentation pathway, resulting in the activation of pathogenic inflammatory and immune responses [26,118]. Indeed, the function of ERAAP (the ERAP1 equivalent in mice) is important for MHC class I peptide processing and presentation, and its destruction leads to an abnormal CD8^+^ T cell response in mice [119]. In addition, mice expressing human *ERAP1* alleles associated with high AS risk were found to generate a different cytotoxic T cell response, when vaccinated with a foreign antigen, than mice expressing a protective human *ERAP1* allele [120]. Taken together, these and other data suggest that dysregulated ERAP1 and ERAP2 activity may lead to altered immunodominance, and modify the activation of cytotoxic T cells [121,122] and NK cells [123], ultimately affecting adaptive and innate immune responses.

### 2.5. Gut Inflammation and Dysbiosis Hypothesis

**Figure 2:**
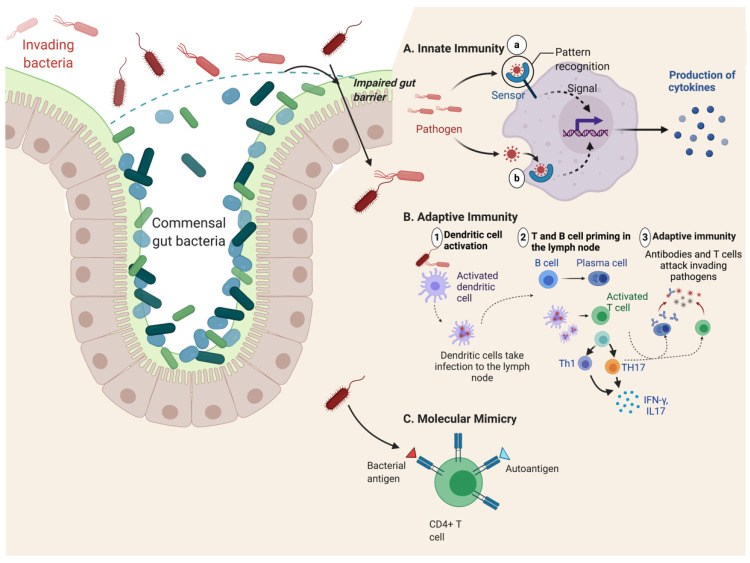
Contribution of gut dysbiosis to autoimmune disease. Due to impaired gut barrier function, pathogenic bacteria can invade the gut lumen and promote overactivation of innate and adaptive immune responses. (**A**) Innate immunity is activated by exposure to bacteria present in the gut tissue and circulation, resulting in excess production of proinflammatory cytokines. (**B**) Bacterial antigens can be presented to CD4+ cells by dendritic cells, leading to the activation of different T cell subtypes (Th1 and Th17) and production of cytokines. B cells are also activated either directly, or through interaction with antigen-presenting dendritic cells, leading to their differentiation into plasma cells and the production of anti-microbial antibodies. (**C**) Bacterial antigens can also provoke autoimmunity by molecular mimicry. Shared sequence similarity between foreign- and self-peptides results in the cross-activation of autoreactive T or B cells by a pathogen-derived antigen. The figure was adapted from “Keystone Gut Microbiota Species Provide Colonization Resistance to Invading Bacteria”, by BioRender.com (2020), retrieved from https://app.biorender.com/biorender-templates. Abbreviations: IL, interleukin; TH-1, T helper type 1; TH-17 T helper type 17.

The most recent hypothesis explaining SpA pathogenesis is the role of HLA-B*27 in shaping the gut microbiome, and HLA-B*27-mediated changes in the gut microbiome associated with disease susceptibility [41]. The gut microbiome is a complex homeostatic ecosystem consisting of trillions of bacteria, and plays important roles in the development of the host`s immune system, in food digestion, and the intestinal epithelial barrier [41,124]. Changes in gut microbiome composition are correlated with autoimmune diseases through mechanisms such as the activation of the immune response, molecular mimicry, and increased intestinal permeability (Figure 2, [41]).

Various studies have shown that the presence of specific changes in the gut microbiome can be responsible for the initiation of autoinflammatory and autoimmune diseases. Such changes include, an increased number of opportunistic pathogens, gut dysbiosis, and a shift in the composition of commensal bacteria [125,126]. Gut dysbiosis could trigger antigenic activation of pathogenic effector cells of the immune system, which in turn facilitate chronic inflammation. Increased gut permeability is a characteristic, and pathogenic pathway, of SpA development (Figure 2). Tight junctions between intestinal epithelial cells are dysregulated, which leads to increased gut permeability and harms mucosal immunity by altering normal regulation of gut microbiota, and the secretion of pro-inflammatory cytokines (Figure 2, [127]). The evidence for this hypothesis is that intestinal permeability is increased in AS patients, and that bacterial by-products have been detected in the synovial fluid of joints of AS patients [128].

A large body of evidence supports a relationship between gut inflammation and SpA. SpA patients have a distinct gut microbiota compared to healthy controls and 60–70% of patients with SpA present microscopic evidence of gut inflammation [129]. In patients, overlap between SpA and inflammatory bowel disease (IBD) is common. For example, 7% of AS patients also have IBD [124]. Conversely, 10–50% of IBD patients develop SpA [124]. In addition, clinical remission of SpA is always associated with normal digestive histology, while the persistence of arthritic symptoms is typically linked to chronic intestinal inflammation [130].

Gut inflammation may be driven by the activation of innate immune cells, such as macrophages, neutrophils, and NK cells [131]. These immune cells recognize pathogenic bacteria (or viruses) through various pattern recognition receptors, leading to the activation of innate immune defense functions, such as phagocytosis, autophagy, and the increased production of pro-inflammatory (TNF-α, IL-1, IL-23, IL-17) cytokines (Figure 2A; [131]). The host genetic background also plays a significant role in gut dysbiosis, leading to a chronic inflammatory state which can promote arthritis [46,132]. Animal, and increasingly also patient, studies show that gut dysbiosis in AS is strongly associated with HLA-B*27 [31,46,133]. HLA-B*27 transgenic rats develop spontaneous spondyloarthropathy, analogous to AS patients [23,134]. However, the development of intestinal or peripheral joint inflammation is prevented in germ-free HLA-B*27 rats [133], but can be restored by re-introduction of specific bacterial taxa [135].

HLA-B*27 may promote gut dysbiosis via direct or indirect mechanisms that involve several processes relevant to disease pathogenesis, such as the activation of adaptive or innate immune responses, and changes in epithelial barrier function. HLA-B*27 transgenic rats exhibit activation of innate immunity and Th17 cell expansion [136]. The altered innate immune response may be linked to the non-antigen-presenting functions of HLA-B*27, including HLA-B*27 misfolding and the induction of an unfolded protein response, as discussed in previous sections. HLA-B*27 may also facilitate the infection, and intracellular survival, of gram-negative bacteria in HLA-B*27-expressing cells [58,137]. Finally, HLA-B*27 may alter cross-presentation of antigens, and thereby modify innate and adaptive immune responses [31,46,133]. However, the precise role of HLA-B*27 in the development of gut dysbiosis, and the mechanisms underlying the linkage between dysbiosis and SpA, remain to be fully understood.

The advent of genomic technologies, and the advancement in bioinformatic analyses, will facilitate the comprehensive understanding of the gut microbiome changes of SpA patients. Recent years have seen tremendous progress in this field [41]. Through various metagenomics studies, the gut microbiome composition of SpA patients was demonstrated to be different from healthy controls. For example, imbalances in *Lachnospiraceae*, *Veillonellaceae*, *Prevotellaceae*, *Porphyromonadaceae,* and *Bacteroides* spp. were reported in patients with AS [126,138,139,140], although no clear microbiome signature emerged from these studies. Deep shotgun sequencing of 211 Chinese individuals further identified 23,709 genes and 12 metagenomic species that were differentially expressed between AS patients and healthy controls [126,138]. These studies reproducibly demonstrated that the gut microbiome undergoes significant changes in its composition, between healthy individuals and AS patients, but also revealed that interpersonal variations in microbiome composition within patient cohorts, replicated in animal models [141], clearly exist. Notably, different gut microbiome compositions were also detected in healthy HLA-B*27 positive and negative individuals, which supports the importance of host genetic background in shaping the gut microbiome [142].

While little is yet know regarding the physiological [142] consequences of specific microbiome imbalances, several studies support a possible immunomodulating role of certain bacterial species in SpA. For example, a metagenomics study analyzed the gut microbiome composition in three cohorts of volunteers: SpA patients, rheumatoid arthritis (RA) patients, and healthy controls [142]. A disease-specific dysbiosis was identified in SpA and RA patients, but not in healthy controls; in particular, significantly increased levels of *Ruminococcus gnavus* were found in SpA patients, and levels of this taxa correlated with IBD severity in this patient cohort [142]. *Ruminococcus gnavus* expansion was also observed in systemic lupus erythematosus (SLE) patients [143] and in patients with Crohn’s disease [144], an inflammatory bowel disease frequently associated with SpA [145]. Significantly, *Ruminococcus gnavus* produces an inflammatory polysaccharide, which induces the secretion of inflammatory cytokines, such as TNFα, by dendritic cells [144]. *Ruminococcus gnavus* expansion may thus contribute to the pathogenesis of SpA, SLE, and Crohn’s disease through the proinflammatory role of a derivative.

Taken together, there is substantial evidence supporting the notion that complex interactions between host genetics, environmental factors, and the gut microbiome drive the pathogenesis of SpA [146]. Pathogenic bacteria, and possibly viruses occurring in the gut of SpA patients, contribute to dysbiosis, and lead to immune dysfunction and modulation of the innate and adaptive immune responses ([126]. The interplay of host genetics and the gut microbiome may further contribute to the development of chronic autoinflammation and autoimmunity.

## 3. Conclusions

**Figure 3:**
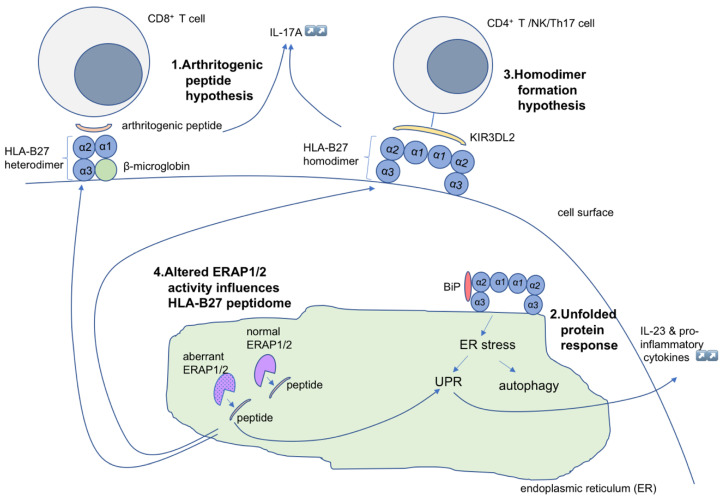
Illustration of the hypothesis for the pathogenetic role of HLA-B*2705 molecules in spondyloarthritis. (1) Arthritogenic peptides displayed by properly folded HLA-B*27 can be recognized by autoreactive CD8^+^ T cells, resulting in inflammation. (2) Misfolded HLA-B*27 chains and binding of BiP causes ER stress and activation of UPR, leading to increased production of IL-23 and other proinflammatory cytokines. (3) Cell surface HLA homodimers interact with CD4^+^ T cells through innate immune receptors, such as KIR3DL2, and facilitate cell-mediated autoimmune responses. (4) Altered ERAP1 activity can result in changes in peptide processing, with pathological consequences. ER, endoplasmic reticulum; ERAP1, ER aminopeptidases (ERAP)1; KIR3DL2, killer immunoglobulin-like receptor; UPR, unfolded protein response.

Despite intensive research, the exact mechanism of SpA pathogenesis and the pathogenic role of HLA-B*27 remain unclear. Five major hypotheses each explain the role of HLA-B*27 in SpA: the arthritogenic peptide hypothesis, the misfolded HLA-B*27 hypothesis, the cell-surface homodimer formation hypothesis, the hypothesis on the malfunctioning of ERAPs, and the hypothesis based on gut microbiome changes. In the previous sections, these hypotheses were described, and it was shown that there is ample evidence to support each of these. It is possible that all, or most, of these mechanisms contribute to disease in individuals. Moreover, taking into account that the frequencies of HLA-B*27 alleles and *ERAP1* polymorphisms are ethnic-specific, it is important to understand that SpA pathogenesis could well be the result of different combinations of these mechanisms in different populations (Figure 3).

The link between these hypotheses could be canonical and non-canonical features of HLA-B*27 and *ERAP1* polymorphisms. In particular, *ERAP1* polymorphisms may contribute to all three models (arthritogenic peptide theory, unfolded protein response, and homodimer formation) that explain how HLA-B*27 causes AS. ERAP1 could contribute to SpA by altering antigen presentation, due to abnormal aminopeptidase activity and dysregulation of peptide processing. Altered rates of peptide trimming, mediated by ERAP1, could lead to cell surface presentation of aberrant peptides for the HLA-B*27 ligand. This facilitates modulation of the immune response through pro-inflammatory cytokines. Recently, *ERAP1* variants have been reported to alter levels of HLA-B*27 free heavy chains on the cell surface. Altered enzyme activity could thus affect the rate at which MHC- peptide complexes fold within the ER and contribute to the UPR and ER stress. The malfunctioning of ERAP1 could facilitate the accumulation of abnormally folded HLA-B*27 in the ER, leading to the activation of the unfolded protein response and a downstream immune response, such as through the upregulation of IL-23.

In conclusion, it is highly likely that more than one of these mechanisms is involved in SpA pathogenesis, and independently or synergistically contributes to the development of autoinflammation or autoimmunity, by exacerbating the pathogenic role of HLA-B*27. This is strengthened by the fact that in each of these hypotheses HLA-B*27 plays a central role.

## References

[1] Zochling J., Brandt J., Braun J. (2005). The current concept of spondyloarthritis with special emphasis on undifferentiated spondyloarthritis. Rheumatology.

[2] Khan M.A. (2013). Polymorphism of HLA-B27: 105 subtypes currently known. Curr. Rheumatol. Rep..

[3] Stolwijk C., Boonen A., van Tubergen A., Reveille J.D. (2012). Epidemiology of spondyloarthritis. Rheum. Dis. Clin. N. Am..

[4] Bakland G., Nossent H.C. (2013). Epidemiology of Spondyloarthritis: A Review. Curr. Rheumatol. Rep..

[5] Braun J., Sieper J. (2007). Ankylosing spondylitis. Lancet.

[6] Gotch F., Rothbard J., Howland K., Townsend A., McMichael A. (1987). Cytotoxic T lymphocytes recognize a fragment of influenza virus matrix protein in association with HLA-A2. Nature.

[7] Townsend A.R.M., Rothbard J., Gotch F.M., Bahadur G., Wraith D., McMichael A.J. (1986). The epitopes of influenza nucleoprotein recognized by cytotoxic T lymphocytes can be defined with short synthetic peptides. Cell.

[8] Colbert R.A., Tran T.M., Layh-Schmitt G. (2014). HLA-B27 misfolding and ankylosing spondylitis. Mol. Immunol..

[9] Hulsmeyer M., Hillig R.C., Volz A., Ruhl M., Schroder W., Saenger W., Ziegler A., Uchanska-Ziegler B. (2002). HLA-B27 subtypes differentially associated with disease exhibit subtle structural alterations. J. Biol. Chem..

[10] Krissinel E., Henrick K. (2007). Inference of macromolecular assemblies from crystalline state. J. Mol. Biol..

[11] Trowsdale J., Knight J.C. (2013). Major histocompatibility complex genomics and human disease. Ann. Rev. Genomics Hum. Genet..

[12] Bjorkman P., Parham P. (1990). Structure, Function, and Diversity of Class I Major Histocompatibility Complex Molecules. Ann. Rev. Biochem..

[13] Crux N., Elahi S. (2017). Human Leukocyte Antigen (HLA) and Immune Regulation: How Do Classical and Non-Classical HLA Alleles Modulate Immune Response to Human Immunodeficiency Virus and Hepatitis C Virus Infections?. Front. Immunol..

[14] McMichael A., Bowness P. (2002). HLA-B27: Natural function and pathogenic role in spondyloarthritis. Arthritis Res..

[15] Reveille J.D., Rich R.R., Fleisher T.A., Shearer W.T., Schroeder H.W., Frew A.J., Weyand C.M. (2019). 57-Spondyloarthritis. Clinical Immunology.

[16] Hill Gaston J.S., Lillicrap M.S. (2003). Arthritis associated with enteric infection. Best Pract. Res. Clin. Rheumatol..

[17] Sieper J., Braun J., Kingsley G.H. (2000). Report on the Fourth International Workshop on Reactive Arthritis. Arthritis Rheum..

[18] Granfors K., Jalkanen S., von Essen R., Lahesmaa-Rantala R., Isomäki O., Pekkola-Heino K., Merilahti-Palo R., Saario R., Isomäki H., Toivanen A. (1989). Yersinia Antigens in Synovial-Fluid Cells from Patients with Reactive Arthritis. N. Engl. J. Med..

[19] Allen R.L., Bowness P., McMichael A. (1999). The role of HLA-B27 in spondyloarthritis. Immunogenetics.

[20] Zhu W., He X., Cheng K., Zhang L., Chen D., Wang X., Qiu G., Cao X., Weng X. (2019). Ankylosing spondylitis: Etiology, pathogenesis, and treatments. Bone Res..

[21] Oldstone M.B.A. (1989). Molecular Mimicry as a Mechanism for the Cause and as a Probe Uncovering Etiologic Agent(S) of Autoimmune-Disease. Curr. Top. Microbiol. Immunol..

[22] Grandon B., Rincheval-Arnold A., Jah N., Corsi J.M., Araujo L.M., Glatigny S., Prevost E., Roche D., Chiocchia G., Guenal I. (2019). HLA-B27 alters BMP/TGFbeta signalling in Drosophila, revealing putative pathogenic mechanism for spondyloarthritis. Ann. Rheum. Dis..

[23] Pedersen S.J., Maksymowych W.P. (2019). The Pathogenesis of Ankylosing Spondylitis: An Update. Curr. Rheumatol. Rep..

[24] Garcia-Montoya L., Gul H., Emery P. (2018). Recent advances in ankylosing spondylitis: Understanding the disease and management. F1000Res.

[25] Martin-Esteban A., Sanz-Bravo A., Guasp P., Barnea E., Admon A., de Castro J.A.L. (2017). Separate effects of the ankylosing spondylitis associated ERAP1 and ERAP2 aminopeptidases determine the influence of their combined phenotype on the HLA-B*27 peptidome. J. Autoimmun..

[26] Babaie F., Mohammadi H., Hemmatzadeh M., Ebrazeh M., Torkamandi S., Yousefi M., Hajaliloo M., Rezaiemanesh A., Salimi S., Salimi R. (2020). Evaluation of ERAP1 gene single nucleotide polymorphisms in immunomodulation of pro-inflammatory and anti-inflammatory cytokines profile in ankylosing spondylitis. Immunol. Lett..

[27] Stoll M.L. (2011). Interactions of the innate and adaptive arms of the immune system in the pathogenesis of spondyloarthritis. Clin. Exp. Rheumatol..

[28] Cusick M.F., Libbey J.E., Fujinami R.S. (2012). Molecular Mimicry as a Mechanism of Autoimmune Disease. Clin. Rev. Allergy Immunol..

[29] Kuon W., Holzhutter H.G., Appel H., Grolms M., Kollnberger S., Traeder A., Henklein P., Weiss E., Thiel A., Lauster R. (2001). Identification of HLA-B27-restricted peptides from the Chlamydia trachomatis proteome with possible relevance to HLA-B27-associated diseases. J. Immunol..

[30] Ramos M., Lopez de Castro J.A. (2002). HLA-B27 and the pathogenesis of spondyloarthritis. Tissue Antigens.

[31] Bowness P. (2015). Hla-B27. Ann. Rev. Immunol..

[32] Appel H., Kuon W., Kuhne M., Wu P.H., Kuhlmann S., Kollnberger S., Thiel A., Bowness P., Sieper J. (2004). Use of HLA-B27 tetramers to identify low-frequency antigen-specific T cells in Chlamydia-triggered reactive arthritis. Arthritis Res. Ther..

[33] Hermann E., Yu D.T.Y., Zumbuschenfelde K.H.M., Fleischer B. (1993). Hla-B27-Restricted Cd8 T-Cells Derived from Synovial-Fluids of Patients with Reactive Arthritis and Ankylosing-Spondylitis. Lancet.

[34] Ugrinovic S., Mertz A., Wu P., Braun J., Sieper J. (1997). A single nonamer from the Yersinia 60-kDa heat shock protein is the target of HLA-B27-restricted CTL response in Yersinia-induced reactive arthritis. J. Immunol..

[35] Scofield R.H., Kurien B., Gross T., Warren W.L., Harley J.B. (1995). HLA-B27 binding of peptide from its own sequence and similar peptides from bacteria: Implications for spondyloarthropathies. Lancet.

[36] Ewing C., Ebringer R., Tribbick G., Geysen H.M. (1990). Antibody activity in ankylosing spondylitis sera to two sites on HLA B27.1 at the MHC groove region (within sequence 65–85), and to a Klebsiella pneumoniae nitrogenase reductase peptide (within sequence 181–199). J. Exp. Med..

[37] Ben Dror L., Barnea E., Beer I., Mann M., Admon A. (2010). The HLA-B*2705 peptidome. Arthritis Rheum..

[38] Fiorillo M.T., Maragno M., Butler R., Dupuis M.L., Sorrentino R. (2000). CD8(+) T-cell autoreactivity to an HLA-B27-restricted self-epitope correlates with ankylosing spondylitis. J. Clin. Investig..

[39] Syrbe U., Sieper J., Rose N.R., Mackay I.R. (2020). Chapter 36-Spondyloarthritides. The Autoimmune Diseases.

[40] Schittenhelm R.B., Sian T.C., Wilmann P.G., Dudek N.L., Purcell A.W. (2015). Revisiting the arthritogenic peptide theory: Quantitative not qualitative changes in the peptide repertoire of HLA-B27 allotypes. Arthritis Rheumatol..

[41] Babaie F., Hasankhani M., Mohammadi H., Safarzadeh E., Rezaiemanesh A., Salimi R., Baradaran B., Babaloo Z. (2018). The role of gut microbiota and IL-23/IL-17 pathway in ankylosing spondylitis immunopathogenesis: New insights and updates. Immunol. Lett..

[42] Mear J.P., Schreiber K.L., Munz C., Zhu X.M., Stevanovic S., Rammensee H.G., Rowland-Jones S.L., Colbert R.A. (1999). Misfolding of HLA-B27 as a result of its B pocket suggests a novel mechanism for its role in susceptibility to spondyloarthropathies. J. Immunol..

[43] Kenna T.J., Robinson P.C., Haroon N. (2015). Endoplasmic reticulum aminopeptidases in the pathogenesis of ankylosing spondylitis. Rheumatology.

[44] Ambarus C.A., Yeremenko N., Baeten D.L. (2018). Altered cytokine expression by macrophages from HLA-B27-positive spondyloarthritis patients without evidence of endoplasmic reticulum stress. Rheumatol. Adv. Pract..

[45] Smith J.A. (2018). Regulation of Cytokine Production by the Unfolded Protein Response; Implications for Infection and Autoimmunity. Front. Immunol..

[46] Busch R., Kollnberger S., Mellins E.D. (2019). HLA associations in inflammatory arthritis: Emerging mechanisms and clinical implications. Nat. Rev. Rheumatol..

[47] Navid F., Layh-Schmitt G., Sikora K.A., Cougnoux A., Colbert R.A. (2018). The Role of Autophagy in the Degradation of Misfolded HLA-B27 Heavy Chains. Arthritis Rheumatol..

[48] Ebringer A. (1983). The Cross-Tolerance Hypothesis, Hla-B27 and Ankylosing-Spondylitis. Rheumatology.

[49] Turner M.J., Delay M.L., Bai S., Klenk E., Colbert R.A. (2007). HLA-B27 up-regulation causes accumulation of misfolded heavy chains and correlates with the magnitude of the unfolded protein response in transgenic rats: Implications for the pathogenesis of spondylarthritis-like disease. Arthritis Rheum..

[50] Delay M.L., Turner M.J., Klenk E.I., Smith J.A., Sowders D.P., Colbert R.A. (2009). HLA-B27 Misfolding and the Unfolded Protein Response Augment Interleukin-23 Production and Are Associated with Th17 Activation in Transgenic Rats. Arthritis Rheum..

[51] Antoniou A.N., Ford S., Taurog J.D., Butcher G.W., Powis S.J. (2004). Formation of HLA-B27 homodimers and their relationship to assembly kinetics. J. Biol. Chem..

[52] Dangoria N.S., DeLay M.L., Kingsbury D.J., Mear J.P., Uchanska-Ziegler B., Ziegler A., Colbert R.A. (2002). HLA-B27 misfolding is associated with aberrant intermolecular disulfide bond formation (dimerization) in the endoplasmic reticulum. J. Biol. Chem..

[53] Tran T.M., Satumtira N., Dorris M.L., May E., Wang A., Furuta E., Taurog J.D. (2004). HLA-B27 in transgenic rats forms disulfide-linked heavy chain oligomers and multimers that bind to the chaperone BiP. J. Immunol..

[54] Turner M.J., Sowders D.P., DeLay M.L., Mohapatra R., Bai S., Smith J.A., Brandewie J.R., Taurog J.D., Colbert R.A. (2005). HLA-B27 misfolding in transgenic rats is associated with activation of the unfolded protein response. J. Immunol..

[55] Tran T.M., Dorris M.L., Satumtira N., Richardson J.A., Hammer R.E., Shang J., Taurog J.D. (2006). Additional human beta(2)-microglobulin curbs HLA-B27 misfolding and promotes arthritis and spondylitis without colitis in male HLA-B27-transgenic rats. Arthritis Rheum..

[56] Campbell E.C., Fettke F., Bhat S., Morley K.D., Powis S.J. (2011). Expression of MHC class I dimers and ERAP1 in an ankylosing spondylitis patient cohort. Immunology.

[57] Ciccia F., Accardo-Palumbo A., Rizzo A., Guggino G., Raimondo S., Giardina A., Cannizzaro A., Colbert R.A., Alessandro R., Triolo G. (2014). Evidence that autophagy, but not the unfolded protein response, regulates the expression of IL-23 in the gut of patients with ankylosing spondylitis and subclinical gut inflammation. Ann. Rheum. Dis..

[58] Antoniou A.N., Lenart I., Kriston-Vizi J., Iwawaki T., Turmaine M., McHugh K., Ali S., Blake N., Bowness P., Bajaj-Elliott M. (2019). Salmonella exploits HLA-B27 and host unfolded protein responses to promote intracellular replication. Ann. Rheum. Dis..

[59] Rezaiemanesh A., Mahmoudi M., Amirzargar A.A., Vojdanian M., Jamshidi A.R., Nicknam M.H. (2017). Ankylosing spondylitis M-CSF-derived macrophages are undergoing unfolded protein response (UPR) and express higher levels of interleukin-23. Mod. Rheumatol..

[60] Taurog J.D., Dorris M.L., Satumtira N., Tran T.M., Sharma R., Dressel R., van den Brandt J., Reichardt H.M. (2009). Spondylarthritis in HLA-B27/human beta2-microglobulin-transgenic rats is not prevented by lack of CD8. Arthritis Rheum..

[61] Boyle L.H., Hill Gaston J.S. (2003). Breaking the rules: The unconventional recognition of HLA-B27 by CD4+ T lymphocytes as an insight into the pathogenesis of the spondyloarthropathies. Rheumatology.

[62] Bowness P., Ridley A., Shaw J., Chan A.T., Wong-Baeza I., Fleming M., Cummings F., McMichael A., Kollnberger S. (2011). Th17 cells expressing KIR3DL2+ and responsive to HLA-B27 homodimers are increased in ankylosing spondylitis. J. Immunol..

[63] Kollnberger S., Bird L., Sun M.-Y., Retiere C., Braud V.M., McMichael A., Bowness P. (2002). Cell-surface expression and immune receptor recognition of HLA–B27 homodimers. Arthritis Rheum..

[64] Chen L., Ridley A., Hammitzsch A., Al-Mossawi M.H., Bunting H., Georgiadis D., Chan A., Kollnberger S., Bowness P. (2015). Silencing or inhibition of endoplasmic reticulum aminopeptidase 1 (ERAP1) suppresses free heavy chain expression and Th17 responses in ankylosing spondylitis. Ann. Rheum. Dis..

[65] Brown M.A. (2018). Solving the pathogenesis of ankylosing spondylitis. Clin. Immunol..

[66] Babaie F., Ebrazeh M., Hemmatzadeh M., Sadat Mohammadi F., Gowhari Shabgah A., Hajaliloo M., Ebrahimi A.A., Shirafkan N., Azizi G., Mohammadi H. (2018). Association analysis of ERAP1 gene single nucleotide polymorphism in susceptibility to ankylosing spondylitis in Iranian population. Immunol. Lett..

[67] Giles J., Shaw J., Piper C., Wong-Baeza I., McHugh K., Ridley A., Li D., Lenart I., Antoniou A.N., DiGleria K. (2012). HLA-B27 homodimers and free H chains are stronger ligands for leukocyte Ig-like receptor B2 than classical HLA class I. J. Immunol..

[68] Chan A.T., Kollnberger S.D., Wedderburn L.R., Bowness P. (2005). Expansion and enhanced survival of natural killer cells expressing the killer immunoglobulin-like receptor KIR3DL2 in spondylarthritis. Arthritis Rheum..

[69] Cauli A., Shaw J., Giles J., Hatano H., Rysnik O., Payeli S., McHugh K., Dessole G., Porru G., Desogus E. (2013). The arthritis-associated HLA-B*27:05 allele forms more cell surface B27 dimer and free heavy chain ligands for KIR3DL2 than HLA-B*27:09. Rheumatology.

[70] Lim Kam Sian T.C.C., Indumathy S., Halim H., Greule A., Cryle M.J., Bowness P., Rossjohn J., Gras S., Purcell A.W., Schittenhelm R.B. (2019). Allelic association with ankylosing spondylitis fails to correlate with human leukocyte antigen B27 homodimer formation. J. Biol. Chem..

[71] Evans D.M., Spencer C.C.A., Pointon J.J., Su Z., Harvey D., Kochan G., Opperman U., Dilthey A., Pirinen M., Stone M.A. (2011). Interaction between ERAP1 and HLA-B27 in ankylosing spondylitis implicates peptide handling in the mechanism for HLA-B27 in disease susceptibility. Nat. Genet..

[72] Mahmoudi M., Jamshidi A.R., Amirzargar A.A., Farhadi E., Nourijelyani K., Fallahi S., Oraei M., Noori S., Nicknam M.H. (2012). Association between Endoplasmic Reticulum Aminopeptidase-1 (ERAP-1) and Susceptibility to Ankylosing Spondylitis in Iran. Iran. J. Allergy Asthma Immunol..

[73] Dashti N., Mahmoudi M., Aslani S., Jamshidi A. (2018). HLA-B*27 subtypes and their implications in the pathogenesis of ankylosing spondylitis. Gene.

[74] Davidson S.I., Wu X., Liu Y., Wei M., Danoy P.A., Thomas G., Cai Q., Sun L., Duncan E., Wang N. (2009). Association of ERAP1, but not IL23R, with ankylosing spondylitis in a Han Chinese population. Arthritis Rheum..

[75] Burton P., Clayton D., Cardon L., Craddock N., Duncanson A., Kwiatkowski D., McCarthy M., Ouwehand W., Samani N., Todd J. (2007). Association scan of 14,500 nonsynonymous SNPs in four diseases identifies autoimmunity variants. Nat. Genet..

[76] Brown M.A. (2007). Breakthroughs in genetic studies of ankylosing spondylitis. Rheumatology.

[77] Michalek M.T., Grant E.P., Gramm C., Goldberg A.L., Rock K.L. (1993). A role for the ubiquitin-dependent proteolytic pathway in MHC class I-restricted antigen presentation. Nature.

[78] Rock K.L., Gramm C., Rothstein L., Clark K., Stein R., Dick L., Hwang D., Goldberg A.L. (1994). Inhibitors of the proteasome block the degradation of most cell proteins and the generation of peptides presented on MHC class I molecules. Cell.

[79] Rock K.L., Reits E., Neefjes J. (2016). Present Yourself! By MHC Class I and MHC Class II Molecules. Trends Immunol..

[80] Chemali M., Radtke K., Desjardins M., English L. (2011). Alternative pathways for MHC class I presentation: A new function for autophagy. Cell Mol. Life Sci..

[81] Van Kaer L., Parekh V.V., Postoak J.L., Wu L. (2019). Role of autophagy in MHC class I-restricted antigen presentation. Mol. Immunol..

[82] Burgevin A., Saveanu L., Kim Y., Barilleau E., Kotturi M., Sette A., van Endert P., Peters B. (2008). A detailed analysis of the murine TAP transporter substrate specificity. PLoS ONE.

[83] van Endert P.M., Riganelli D., Greco G., Fleischhauer K., Sidney J., Sette A., Bach J.F. (1995). The peptide-binding motif for the human transporter associated with antigen processing. J. Exp. Med..

[84] Saric T., Chang S.C., Hattori A., York I.A., Markant S., Rock K.L., Tsujimoto M., Goldberg A.L. (2002). An IFN-gamma-induced aminopeptidase in the ER, ERAP1, trims precursors to MHC class I-presented peptides. Nat. Immunol..

[85] Lopez de Castro J.A. (2018). How ERAP1 and ERAP2 Shape the Peptidomes of Disease-Associated MHC-I Proteins eCollection 2018. Front. Immunol..

[86] Cui X.L., Hawari F., Alsaaty S., Lawrence M., Combs C.A., Geng W.D., Rouhani F.N., Miskinis D., Levine S.J. (2002). Identification of ARTS-1 as a novel TNFR1-binding protein that promotes TNFR1 ectodomain shedding. J. Clin. Investig..

[87] Haroon N., Tsui F., Chiu B., Tsui H., Inman R. (2010). Serum Cytokine Receptors in Ankylosing Spondylitis: Relationship to Inflammatory Markers and Endoplasmic Reticulum Aminopeptidase Polymorphisms. J. Rheumatol..

[88] Reveille J.D. (2012). Genetics of spondyloarthritis—Beyond the MHC. Nat. Rev. Rheumatol..

[89] York I.A., Chang S.C., Saric T., Keys J.A., Favreau J.M., Goldberg A.L., Rock K.L. (2002). The ER aminopeptidase ERAP1 enhances or limits antigen presentation by trimming epitopes to 8–9 residues. Nat. Immunol..

[90] Andres A.M., Dennis M.Y., Kretzschmar W.W., Cannons J.L., Lee-Lin S.Q., Hurle B., Program N.C.S., Schwartzberg P.L., Williamson S.H., Bustamante C.D. (2010). Balancing selection maintains a form of ERAP2 that undergoes nonsense-mediated decay and affects antigen presentation. PLoS Genet..

[91] Paladini F., Fiorillo M.T., Tedeschi V., Mattorre B., Sorrentino R. (2020). The Multifaceted Nature of Aminopeptidases ERAP1, ERAP2, and LNPEP: From Evolution to Disease. Front. Immunol..

[92] Chang S.C., Momburg F., Bhutani N., Goldberg A.L. (2005). The ER aminopeptidase, ERAP1, trims precursors to lengths of MHC class I peptides by a “molecular ruler” mechanism. Proc. Natl. Acad. Sci. USA.

[93] Keidel S., Chen L., Pointon J., Wordsworth P. (2013). ERAP1 and ankylosing spondylitis. Curr. Opin. Immunol..

[94] Kirino Y., Bertsias G., Ishigatsubo Y., Mizuki N., Tugal-Tutkun I., Seyahi E., Ozyazgan Y., Sacli F.S., Erer B., Inoko H. (2013). Genome-wide association analysis identifies new susceptibility loci for Behcet’s disease and epistasis between HLA-B*51 and ERAP1. Nat. Genet..

[95] Strange A., Capon F., Spencer C.C.A., Knight J., Weale M.E., Allen M.H., Barton A., Band G., Genetic Analysis of Psoriasis Consortium, the Wellcome Trust Case Control Consortium 2 (2010). A genome-wide association study identifies new psoriasis susceptibility loci and an interaction between HLA-C and ERAP1. Nat. Genet..

[96] Yin X., Low H.Q., Wang L., Li Y., Ellinghaus E., Han J., Estivill X., Sun L., Zuo X., Shen C. (2015). Genome-wide meta-analysis identifies multiple novel associations and ethnic heterogeneity of psoriasis susceptibility. Nat. Commun..

[97] Tsoi L.C., Spain S.L., Knight J., Ellinghaus E., Stuart P.E., Capon F., Ding J., Li Y., Tejasvi T., Gudjonsson J.E. (2012). Identification of 15 new psoriasis susceptibility loci highlights the role of innate immunity. Nat. Genet..

[98] Robinson P.C., Costello M.E., Leo P., Bradbury L.A., Hollis K., Cortes A., Lee S., Joo K.B., Shim S.C., Weisman M. (2015). ERAP2 is associated with ankylosing spondylitis in HLA-B27-positive and HLA-B27-negative patients. Ann. Rheum. Dis..

[99] Cortes A., Hadler J., Pointon J.P., Robinson P.C., Karaderi T., Leo P., Cremin K., Pryce K., Harris J., International Genetics of Ankylosing Spondylitis Consortium (IGAS) (2013). Identification of multiple risk variants for ankylosing spondylitis through high-density genotyping of immune-related loci. Nat. Genet..

[100] Seregin S.S., Rastall D.P., Evnouchidou I., Aylsworth C.F., Quiroga D., Kamal R.P., Godbehere-Roosa S., Blum C.F., York I.A., Stratikos E. (2013). Endoplasmic reticulum aminopeptidase-1 alleles associated with increased risk of ankylosing spondylitis reduce HLA-B27 mediated presentation of multiple antigens. Autoimmunity.

[101] Kochan G., Krojer T., Harvey D., Fischer R., Chen L., Vollmar M., von Delft F., Kavanagh K.L., Brown M.A., Bowness P. (2011). Crystal structures of the endoplasmic reticulum aminopeptidase-1 (ERAP1) reveal the molecular basis for N-terminal peptide trimming. Proc. Natl. Acad. Sci. USA.

[102] McHugh K., Bowness P. (2012). The link between HLA-B27 and SpA—New ideas on an old problem. Rheumatology.

[103] Saveanu L., van Endert P. (2012). The role of insulin-regulated aminopeptidase in MHC class I antigen presentation. Front. Immunol..

[104] Li L., Batliwala M., Bouvier M. (2019). ERAP1 enzyme-mediated trimming and structural analyses of MHC I—Bound precursor peptides yield novel insights into antigen processing and presentation. J. Biol. Chem..

[105] Tang Y., Yang P., Wang F., Xu H., Zong S.Y. (2018). Association of polymorphisms in ERAP1 and risk of ankylosing spondylitis in a Chinese population. Gene.

[106] Wisniewski A., Kasprzyk S., Majorczyk E., Nowak I., Wilczynska K., Chlebicki A., Zon-Giebel A., Kusnierczyk P. (2019). ERAP1-ERAP2 haplotypes are associated with ankylosing spondylitis in Polish patients. Hum. Immunol..

[107] Paladini F., Fiorillo M.T., Tedeschi V., D’Otolo V., Piga M., Cauli A., Mathieu A., Sorrentino R. (2019). The rs75862629 minor allele in the endoplasmic reticulum aminopeptidases intergenic region affects human leucocyte antigen B27 expression and protects from ankylosing spondylitis in Sardinia. Rheumatology.

[108] Lopez de Castro J.A., Alvarez-Navarro C., Brito A., Guasp P., Martin-Esteban A., Sanz-Bravo A. (2016). Molecular and pathogenic effects of endoplasmic reticulum aminopeptidases ERAP1 and ERAP2 in MHC-I-associated inflammatory disorders: Towards a unifying view. Mol. Immunol..

[109] Vitulano C., Tedeschi V., Paladini F., Sorrentino R., Fiorillo M.T. (2017). The interplay between HLA-B27 and ERAP1/ERAP2 aminopeptidases: From anti-viral protection to spondyloarthritis. Clin. Exp. Immunol..

[110] York I.A., Brehm M.A., Zendzian S., Towne C.F., Rock K.L. (2006). Endoplasmic reticulum aminopeptidase 1 (ERAP1) trims MHC class I-presented peptides in vivo and plays an important role in immunodominance. Proc. Natl. Acad. Sci. USA.

[111] Rastall D.P., Aldhamen Y.A., Seregin S.S., Godbehere S., Amalfitano A. (2014). ERAP1 functions override the intrinsic selection of specific antigens as immunodominant peptides, thereby altering the potency of antigen-specific cytolytic and effector memory T-cell responses. Int. Immunol..

[112] Collins E.J., Garboczi D.N., Wiley D.C. (1994). Three-dimensional structure of a peptide extending from one end of a class I MHC binding site. Nature.

[113] Probst-Kepper M., Hecht H.J., Herrmann H., Janke V., Ocklenburg F., Klempnauer J., van den Eynde B.J., Weiss S. (2004). Conformational restraints and flexibility of 14-meric peptides in complex with HLA-B*3501. J. Immunol..

[114] Tynan F.E., Burrows S.R., Buckle A.M., Clements C.S., Borg N.A., Miles J.J., Beddoe T., Whisstock J.C., Wilce M.C., Silins S.L. (2005). T cell receptor recognition of a ‘super-bulged’ major histocompatibility complex class I-bound peptide. Nat. Immunol..

[115] Robinson P.C., Lau E., Keith P., Lau M.C., Thomas G.P., Bradbury L.A., Brown M.A., Kenna T.J. (2015). ERAP2 functional knockout in humans does not alter surface heavy chains or HLA-B27, inflammatory cytokines or endoplasmic reticulum stress markers. Ann. Rheum. Dis..

[116] Lorente E., Fontela M.G., Barnea E., Martin-Galiano A.J., Mir C., Galocha B., Admon A., Lauzurica P., Lopez D. (2020). Modulation of Natural HLA-B*27:05 Ligandome by Ankylosing Spondylitis-associated Endoplasmic Reticulum Aminopeptidase 2 (ERAP2). Mol. Cell. Proteomics.

[117] Lorente E., Redondo-Anton J., Martin-Esteban A., Guasp P., Barnea E., Lauzurica P., Admon A., Lopez de Castro J.A. (2019). Substantial Influence of ERAP2 on the HLA-B*40:02 Peptidome: Implications for HLA-B*27-Negative Ankylosing Spondylitis. Mol. Cell. Proteomics.

[118] Chen B., Li D., Xu W. (2013). Association of ankylosing spondylitis with HLA-B27 and ERAP1: Pathogenic role of antigenic peptide. Med. Hypotheses.

[119] Hammer G.E., Gonzalez F., Champsaur M., Cado D., Shastri N. (2006). The aminopeptidase ERAAP shapes the peptide repertoire displayed by major histocompatibility complex class I molecules. Nat. Immunol..

[120] Rastall D.P.W., Alyaquob F.S., O’Connell P., Pepelyayeva Y., Peters D., Godbehere-Roosa S., Pereira-Hicks C., Aldhamen Y.A., Amalfitano A. (2017). Mice expressing human ERAP1 variants associated with ankylosing spondylitis have altered T-cell repertoires and NK cell functions, as well as increased in utero and perinatal mortality. Int. Immunol..

[121] Garboczi D.N., Ghosh P., Utz U., Fan Q.R., Biddison W.E., Wiley D.C. (1996). Structure of the complex between human T-cell receptor, viral peptide and HLA-A2. Nature.

[122] Jardetzky T. (1997). Not just another Fab: The crystal structure of a TcR-MHC-peptide complex. Structure.

[123] Brooks A.G., Boyington J.C., Sun P.D. (2000). Natural killer cell recognition of HLA class I molecules. Rev. Immunogenet..

[124] Gill T., Asquith M., Brooks S.R., Rosenbaum J.T., Colbert R.A. (2018). Effects of HLA-B27 on Gut Microbiota in Experimental Spondyloarthritis Implicate an Ecological Model of Dysbiosis. Arthritis Rheumatol..

[125] Sartor R.B., Wu G.D. (2017). Roles for Intestinal Bacteria, Viruses, and Fungi in Pathogenesis of Inflammatory Bowel Diseases and Therapeutic Approaches. Gastroenterology.

[126] Rizzo A., Ferrante A., Guggino G., Ciccia F. (2017). Gut inflammation in spondyloarthritis. Best Pract. Res. Clin. Rheumatol..

[127] Reid G., Younes J.A., Van der Mei H.C., Gloor G.B., Knight R., Busscher H.J. (2011). Microbiota restoration: Natural and supplemented recovery of human microbial communities. Nat. Rev. Microbiol..

[128] Mielants H., Veys E.M., De Vos M., Cuvelier C. (1992). Increased intestinal permeability in ankylosing spondylitis. Gut.

[129] Van Praet L., Jacques P., Van den Bosch F., Elewaut D. (2012). The transition of acute to chronic bowel inflammation in spondyloarthritis. Nat. Rev. Rheumatol..

[130] Cypers H., Van Praet L., Varkas G., Elewaut D. (2014). Relevance of the gut/joint axis for the management of spondyloarthritis in daily clinical practice. Curr. Opin. Rheumatol..

[131] Gill T., Asquith M., Rosenbaum J.T., Colbert R.A. (2015). The intestinal microbiome in spondyloarthritis. Curr. Opin. Rheumatol..

[132] Ombrello M.J., Remmers E.F., Tachmazidou I., Grom A., Foell D., Haas J.P., Martini A., Gattorno M., Ozen S., Prahalad S. (2015). HLA-DRB1*11 and variants of the MHC class II locus are strong risk factors for systemic juvenile idiopathic arthritis. Proc. Natl. Acad. Sci. USA.

[133] Taurog J.D., Chhabra A., Colbert R.A. (2016). Ankylosing Spondylitis and Axial Spondyloarthritis. N. Engl. J. Med..

[134] Lin P., Bach M., Asquith M., Lee A.Y., Akileswaran L., Stauffer P., Davin S., Pan Y., Cambronne E.D., Dorris M. (2014). HLA-B27 and human beta2-microglobulin affect the gut microbiota of transgenic rats. PLoS ONE.

[135] Rath H.C., Herfarth H.H., Ikeda J.S., Grenther W.B., Hamm T.E., Balish E., Taurog J.D., Hammer R.E., Wilson K.H., Sartor R.B. (1996). Normal luminal bacteria, especially Bacteroides species, mediate chronic colitis, gastritis, and arthritis in HLA-B27/human beta2 microglobulin transgenic rats. J. Clin. Investig..

[136] Asquith M.J., Stauffer P., Davin S., Mitchell C., Lin P., Rosenbaum J.T. (2016). Perturbed Mucosal Immunity and Dysbiosis Accompany Clinical Disease in a Rat Model of Spondyloarthritis. Arthritis Rheumatol..

[137] Ansalone C., Utriainen L., Milling S., Goodyear C.S. (2017). Role of Gut Inflammation in Altering the Monocyte Compartment and Its Osteoclastogenic Potential in HLA-B27-Transgenic Rats. Arthritis Rheumatol..

[138] Wen C., Zheng Z., Shao T., Liu L., Xie Z., Le Chatelier E., He Z., Zhong W., Fan Y., Zhang L. (2017). Quantitative metagenomics reveals unique gut microbiome biomarkers in ankylosing spondylitis. Genome Biol..

[139] Costello M.E., Ciccia F., Willner D., Warrington N., Robinson P.C., Gardiner B., Marshall M., Kenna T.J., Triolo G., Brown M.A. (2015). Brief Report: Intestinal Dysbiosis in Ankylosing Spondylitis. Arthritis Rheumatol..

[140] Zhang L., Hu Y., Xu Y., Li P., Ma H., Li X., Li M. (2019). The correlation between intestinal dysbiosis and the development of ankylosing spondylitis. Microb. Pathog..

[141] Gill T., Brooks S., Rosenbaum J., Asquith M., Colbert R. (2019). Novel Inter-omic Analysis Reveals Relationships Between Diverse Gut Microbiota and Host Immune Dysregulation in HLA-B27-Induced Experimental Spondyloarthritis. Arthritis Rheumatol..

[142] Breban M., Tap J., Leboime A., Said-Nahal R., Langella P., Chiocchia G., Furet J.P., Sokol H. (2017). Faecal microbiota study reveals specific dysbiosis in spondyloarthritis. Ann. Rheum. Dis..

[143] Azzouz D., Omarbekova A., Heguy A., Schwudke D., Gisch N., Rovin B.H., Caricchio R., Buyon J.P., Alekseyenko A.V., Silverman G.J. (2019). Lupus nephritis is linked to disease-activity associated expansions and immunity to a gut commensal. Ann. Rheum. Dis..

[144] Henke M.T., Kenny D.J., Cassilly C.D., Vlamakis H., Xavier R.J., Clardy J. (2019). Ruminococcus gnavus, a member of the human gut microbiome associated with Crohn’s disease, produces an inflammatory polysaccharide. Proc. Natl. Acad. Sci. USA.

[145] Levine J.S., Burakoff R. (2011). Extraintestinal manifestations of inflammatory bowel disease. Gastroenterol. Hepatol..

[146] Yin J., Sternes P.R., Wang M., Song J., Morrison M., Li T., Zhou L., Wu X., He F., Zhu J. (2020). Shotgun metagenomics reveals an enrichment of potentially cross-reactive bacterial epitopes in ankylosing spondylitis patients, as well as the effects of TNFi therapy upon microbiome composition. Ann. Rheum. Dis..

